# Optimizing Screening Performance for the Risk of Hyperoxaluria and Urolithiasis Using the Urinary Oxalate/Creatinine Ratio: A Retrospective Analysis

**DOI:** 10.1016/j.euros.2025.03.003

**Published:** 2025-03-28

**Authors:** Pierre Letourneau, Lara Cabezas, Aurélie De Mul, Nadia Abid, Christelle Machon, Cécile Poussineau, Cécile Acquaviva, Justine Bacchetta, Laurence Derain-Dubourg, Sandrine Lemoine

**Affiliations:** a Department of Nephrology, Dialysis, Hypertension, and Renal Function Exploration, Edouard Herriot Hospital, Hospices Civils de Lyon, Lyon, France; b Department of Urology, Edouard Herriot Hospital, Hospices Civils de Lyon, Lyon, France; c Department of Biochemistry and Molecular Biology, Hospices Civils de Lyon, Lyon, France; d Reference Center for Rare Renal Diseases, Pediatric Nephrology-Rheumatology-Dermatology Unit, Hospices Civils de Lyon, Lyon, France; e INSERM CARMEN 1060, IRIS Team, INSERM 1033, University of Lyon, Lyon, France

**Keywords:** Bariatric surgery, Bypass enteric hyperoxaluria, Hyperoxaluria, Kidney stones, Oxalate, Oxalate excretion, Oxaluria, Urolithiasis

## Abstract

**Background and objective:**

The risk of chronic kidney disease (CKD) and nephrolithiasis increases with higher levels of oxalate excretion in 24-h urine, warranting monitoring in specific populations, especially after malabsorptive bariatric surgery. However, implementation of systematic 24-h urine collection is challenging, so there is a need for alternative screening methods.

**Methods:**

Using retrospective data from patients evaluated for the risk of urolithiasis, we assessed different thresholds for the urinary oxalate/creatinine (UOx/Creat) ratio to optimize the screening performance for hyperoxaluria diagnosis and urolithiasis risk using lithogenic risk surrogates.

**Key findings and limitations:**

Among 1264 patients referred for urolithiasis, 38% were excluded because urine collection was considered incomplete. The remaining 786 individuals were included in our analysis, of whom 16% exhibited hyperoxaluria. A UOx/Creat screening threshold between 35 and 45 μmol/mmol demonstrated good performance, depending on the clinical weighting for false-negative versus true-positive results and the cost/benefit ratio.

**Conclusions and clinical implications:**

The UOx/Creat ratio is a promising tool in screening for hyperoxaluria-related complications. However, future research is needed to validate its performance and address the limitations identified to confirm its clinical relevance and effectiveness.

**Patient summary:**

Our study shows that a simple test to measure the ratio of oxalate to creatinine in urine can help in identifying people at risk of kidney stones, especially for patients who have had weight-loss surgery. The test showed good screening performance, but more research is needed to confirm our findings.

## Take Home Message

The urinary oxalate/creatinine ratio is a promising tool for screening for hyperoxaluria-related complications such as urolithiasis in various populations. Further research is necessary to overcome the limitations identified and validate the performance of oxalate/creatinine thresholds in an external cohort.

## 1 Introduction

The risk of urolithiasis and incident chronic kidney disease (CKD) rises with higher oxalate excretion in 24-h urine [1,2]. Therefore, this parameter should be monitored in specific conditions, such as primary hyperoxaluria, intestinal inflammatory diseases, short bowel syndrome, and malabsorptive bariatric surgery, in which urinary oxalate is markedly elevated [3]. All of these pathological conditions can lead to urolithiasis, oxalate nephropathy, and, for some patients, systemic oxalate accumulation. Although the risk of nephropathy in primary hyperoxaluria is largely known, nephrologists probably underestimate this risk in malabsorptive conditions. Recent reports highlighted a twofold higher risk of kidney stones in a bypass population [4], elevated incidence of oxalate nephropathy and kidney stones in enteric hyperoxaluria [3,5,6], and association of oxaluria with CKD progression [7]. Considering these associations, it is crucial to identify individuals at the highest risk of hyperoxaluria within these specific populations. Early identification can help in preventing the development of kidney stones and CKD, as these individuals would benefit from nephrological intervention and tailored dietary advice to mitigate these risks [8].

Assessment of lithogenic risk traditionally relies on factors such as fluid intake, the presence, and severity of enteric hyperoxaluria, other abnormalities in 24-h urine, and identification of crystals in urine [9]. While 24-hour urine collection is the optimal specimen for evaluating hyperoxaluria and assessing the risk of kidney stone formation, implementation across all hyperoxaluria populations poses practical challenges. The urinary oxalate/creatinine ratio (UOx/Creat) has been suggested as a measure for hyperoxaluria detection, but limited data are available for adults excluding primary hyperoxaluria. Depending on the laboratory and the pathological condition, the analytical method used for quantification of oxaluria varies (enzymatic assays, liquid chromatography and gas chromatography coupled to mass spectrometry). Considering the variability among analytical methods, normal values depend on the method used. In addition, agreement between the UOx/Creat ratio and 24-h urine assessment is not consistently robust and the performance of the UOx/Creat ratio in predicting urolithiasis risk is not well established [[10], [11], [12], [13], [14]]. Technical notes from suppliers of enzyme kits for oxaluria determination do not indicate normal values for the UOx/Creat ratio. A threshold of 80 μmol/mmol has been proposed for a gas chromatography-mass spectrometry method for diagnosing primary oxaluria [15]. However, this threshold is significantly higher than the 34 and 45 μmol/mmol values recommended in European guidelines and is not adapted for urolithiasis risk assessment and oxaluria determined with an enzymatic method [16].

Hence, our primary objective was to evaluate the diagnostic performance of the UOx/Creat ratio in identifying individuals with 24-hour hyperoxaluria and those at elevated risk of urolithiasis. We also sought to determine suitable screening thresholds for this ratio.

## 2 Patients and methods

### 2.1 Study population

We evaluated thresholds for the UOx/Creat ratio using retrospective data collected in a tertiary nephrology hospital with a stone clinic (Hospices Civils de Lyon, Lyon, France). We included all patients evaluated from 2014 to 2023 for urolithiasis risk who were aged >18 yr, including those with short bowel syndrome, bariatric surgery, dietetic lithiasis, and primary hyperoxaluria. We excluded patients with multiple visits and those for whom important data were missing.

### 2.2 Study ethics

All procedures were conducted in accordance with institutional ethics standards and, the 2013 Declaration of Helsinki and its later amendments, or comparable ethics standards. Appropriate informed consent was obtained from each participant or his/her legal representative. The consent form included information on the procedure and the possibility of later use of the data for research purposes. According to French law applicable at the time of the study, an observational study that does not change the routine management for patients did not need to be declared or submitted to a research ethics board (Loi Huriet-Sérusclat 88–1138, December 20, 1988 and subsequent amendments; http://www.chu-toulouse.fr/IMG/pdf/loihuriet.pdf).

### 2.3 Biochemical analysis

Usual blood metabolic analyses were conducted on 24-h urine samples to measure determinants of urinary supersaturation. Urinary oxalate was quantified using an enzyme method (Trinity Biotech oxalate kit). In brief, after sample purification using activated charcoal, oxalate is oxidized to carbon dioxide and hydrogen peroxide by oxalate oxidase. The hydrogen peroxide reacts with 3-methyl-2-benzothiazolinone hydrazine and 3-(dimethylamino)-benzoic acid in the presence of peroxidase to produce a colored indamine. The absorbance measured at 590 nm after this reaction is directly proportional to the concentration of urinary oxalate. The method applied was validated according to the ISO 15189 standard. The kit demonstrated good analytical performance, with coefficients of variation <0.9% and <2.5% for within-day and between-day variability, respectively. Complete 24-h urine collection was defined as a 24-h creatinine result within the theoretical creatinine range ±20% estimated as the average of formulas D and E of Ix et al [17] and the Walser formula [18]. Hyperoxaluria diagnosis was confirmed when oxaluria in a complete 24-h urine sample was above the upper limit of normal (expected range 40–320 μmol/24 h for women and 80–490 μmol/24 h for men with the kit used). The estimated glomerular filtration rate (eGFR) was assessed using CKD Epidemiology Collaboration equation [19].

### 2.4 Surrogate markers for urolithiasis risk

Robertson [20] developed a comprehensive method for assessing the biochemical risk of kidney stone formation (probability of stone formation, PSF) that uses risk curves derived from frequency distributions for seven risk factors measured in 24-h urine samples from numerous idiopathic stone-formers and control counterparts. Specifically, PSF(CaOx) is a score for predicting calcium oxalate stone formation, and PSF(CaOx) >0.5 effectively distinguishes individuals prone to stone formation. PSF(CaOx) also provides insights into the potential severity of the disorder in an individual, as indicated by the frequency of stone episodes experienced per year. Therefore, we assessed the biochemical risk of stone formation using the PSF(CaOx) risk model, with a score >0.5 considered as predictive of urolithiasis risk [20]. We also used crystalluria as a surrogate for high risk of kidney stone formation according to the method described by Daudon et al [21]. Crystalluria analysis was conducted on fresh urine samples (fasting sample collected the day after 24-h urine collection) using a phase-contrast microscope equipped with a polarizing light device. After homogenization, the urine sample was deposited on a Kova Glasstic slide and any crystals present were identified and counted.

### 2.5 Statistics

All statistical analyses were performed using R v4.2.1 (https://www.r-project.org/). Data are reported as the median and interquartile range (IQR), mean and standard deviation (SD), or frequency and percentage. QQ plots and the Shapiro-Wilk test were used to assess the normality of distribution when needed. Receiver operating characteristic (ROC) curves were generated using easyROC v1.3.1 [22]. We used a generalized Youden index that incorporates a cost/benefit analysis to guide threshold selection, for which the cost equivalent for a false-negative tests was assigned a value five times that for a false-positive test. All differences were considered significant at *p* < 0.05.

## 3 Results

### 3.1 Study population

**Fig. 1:**
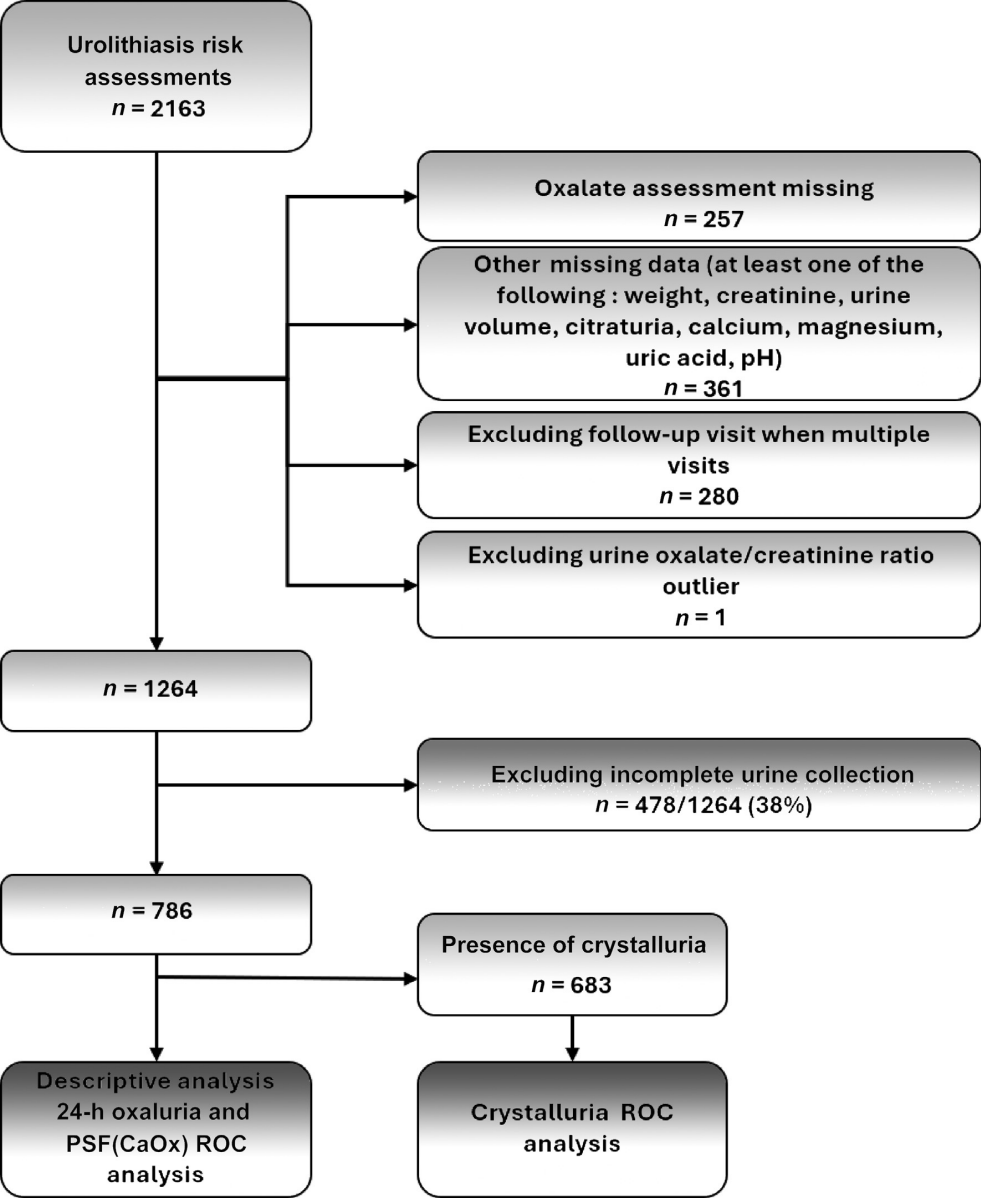
Study flowchart.

We screened 2163 clinic visits for urolithiasis risk assessment and identified 1264 unique visits with complete data. Of the 1264 adult patients identified (Fig. 1) 38% were excluded because urine collection was considered incomplete according to established criteria, resulting in a study population of 786 individuals (359 females, 427 males; median age 48 yr, median eGFR 97 ml/min/1.73 m^2^). The prevalence of hyperoxaluria was 16% overall (females of 22% vs males 11%). Median 24-h oxaluria was 561 μmol/d (IQR 438–771) in the hyperoxaluria group and 245 μmol/d (IQR 185–309) in the group without hyperoxaluria. The hyperoxaluria group had a higher positive whewellite crystalluria rate and higher PSF(CaOx) score (Table 1).

**Table 1:** Patient characteristics by hyperoxaluria status

Parameter	No hyperoxaluria (*n* = 660)	Hyperoxaluria (*n* = 126)	*p* value^a^
Median age, yr (IQR)	47 (34–59)	52 (40–58)	0.027
Median body mass index, kg/m^2^ (IQR)	24.7 (21.8–28.1)	26.3 (23.1–31.2)	0,001
Female, *n* (%)	281 (43)	78 (62)	<0.001
Median plasma creatinine, μmol/l (IQR)	75 (63–87)	67 (57–82)	0.003
Median eGFR, ml/min/1.73m^2^ (IQR)	97 (84–110)	98 (79–107)	0.4
Median urinary oxalate, μmol/d (IQR)	245 (185–309)	561 (438–771)	<0.001
Median urinary oxalate/creatinine ratio, μmol/mmol (IQR)	21 (16–27)	53 (40–75)	<0.001
Crystalluria results, *n* (%)			<0.001
No crystals	481 (83)	81 (74)	
Weddellite crystals	84 (15)	14 (13)	
Whewellite crystals	13 (2.2)	15 (14)	
Data not available	82	16	
Median PSF(CaOx) score (IQR)	0.02 (0.02–0.04)	0.22 (0.07–0.91)	<0.001
PSF(CaOx) >0.5, *n* (%)	12 (1.8)	46 (37)	<0.001
PSF(CaOx) >0.9, *n* (%)	7 (1.1)	32 (25)	<0.001

eGFR = estimated glomerular filtration rate (Chronic Kidney Disease Epidemiology Consortium equation); IQR = interquartile range; PSF(CaOx) = probability of calcium oxalate stone formation.

a Wilcoxon rank-sum test or Pearson’s *χ*^2^ test, as appropriate.

### 3.2 Diagnostic performance of UOx/Creat for hyperoxaluria

**Fig. 2:**
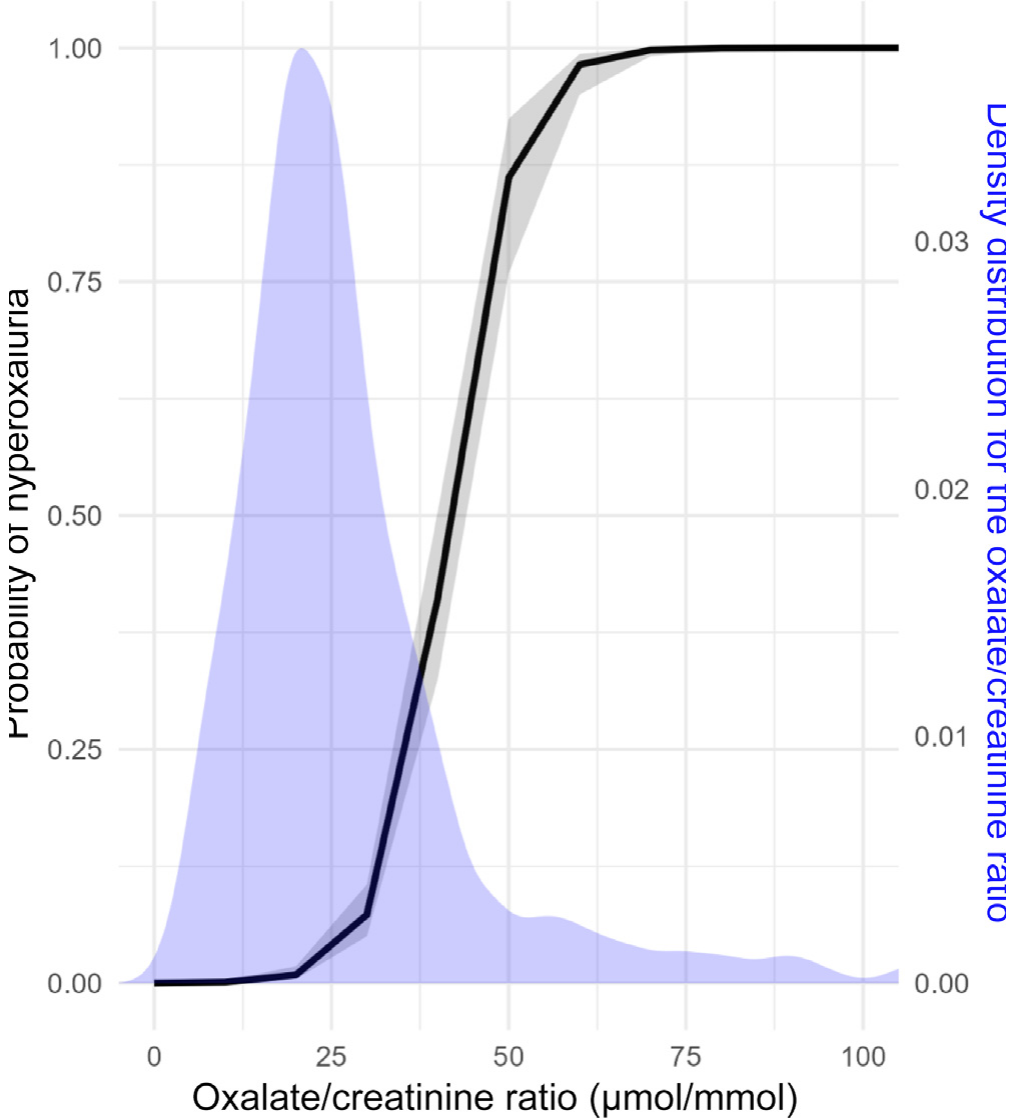
Probability of hyperoxaluria as a function of the urinary oxalate/creatinine ratio for the overall cohort, overlaid with the density distribution.

ROC analysis of UOx/Creat for hyperoxaluria diagnosis in the overall population yielded an area under the ROC curve (AUC) of 0.97 (95% confidence interval [CI] 0.96–0.98; Fig. 2). Table 2 shows the clinical implications of different UOx/Creat thresholds, including the percentage of patients requiring confirmatory testing and rates of hyperoxaluria cases missed (false negatives) and detected (true positives). UOx/Creat ratios between 35 and 45 μmol/mmol demonstrated good screening performance, with a false-negative rate ranging from 2% to 6.1% and 10.8–20% of patients requiring confirmatory testing.

**Table 2:** Clinical implications of different UOx/Creat thresholds for hyperoxaluria

UOx/Creat threshold	Confirmatory test needed (%)	Hyperoxaluria result (%)			
		FN	TP	TN	FP
30 μmol/mmol	29.5	0.9	15.1	69.6	14.4
32.5 μmol/mmol	24.4	1.3	14.8	74.3	9.7
35 μmol/mmol	20.0	2.0	14.0	77.9	6.0
37.5 μmol/mmol	16.8	3.1	13.0	80.0	3.8
40 μmol/mmol	14.4	4.1	12.0	81.6	2.4
42.5 μmol/mmol	12.0	5.1	10.9	83.0	1.0
45 μmol/mmol	10.8	6.1	9.9	83.1	0.9
47.5 μmol/mmol	9.9	6.6	9.4	83.5	0.5
50 μmol/mmol	9.0	7.4	8.7	83.6	0.4
60 μmol/mmol	6.5	9.7	6.4	83.8	0.1
70 μmol/mmol	4.7	11.5	4.6	83.8	0.1
80 μmol/mmol	3.4	12.6	3.4	84.0	0.0

FN = false negative; FP = false positive; TN = true negative; TP = true positive; UOx/Creat = urinary oxalate/creatinine ratio.

**Fig. 3:**
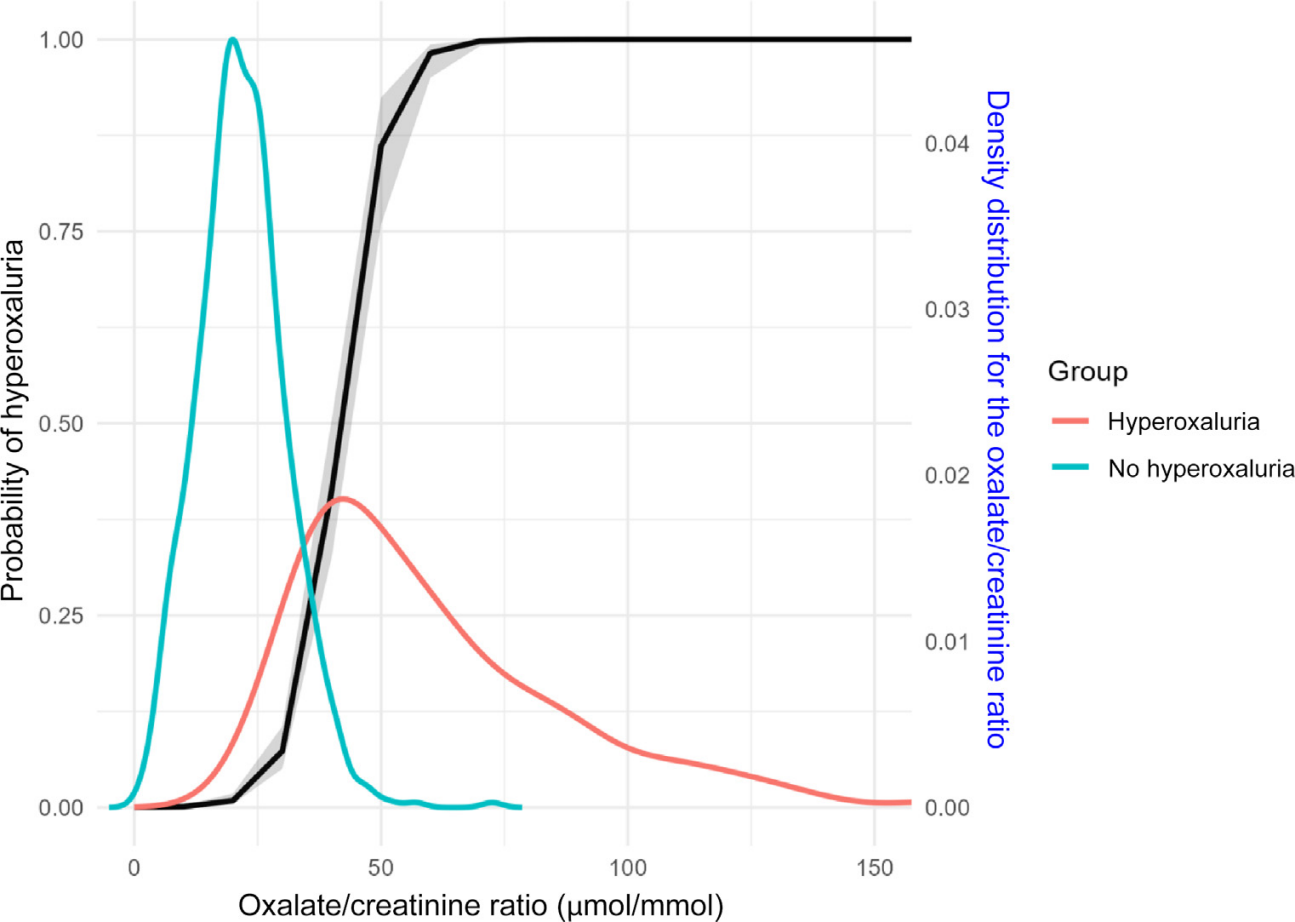
Probability of hyperoxaluria as a function of the urinary oxalate/creatinine ratio for the groups with and without hyperoxaluria, overlaid with the overall density distribution.

AUC values for both males (0.98, 95% CI 0.97–0.99) and females (0.95, 95% CI 0.93–0.98) were good, with comparable performance across various UOx/Creat thresholds (data not shown). Fig. 2 shows the relationship between the UOx/Creat ratio and the probability of hyperoxaluria, along with the density distribution for the UOx/Creat ratio, for the overall population. Fig. 3 shows the results stratified by hyperoxaluria status, which indicate that the probability of hyperoxaluria is high for UOx/Creat >50 μmol/mmol. A calibration plot of the model is also provided in Supplementary Fig. 1.

### 3.3 Diagnostic performance of UOx/Creat for PSF(CaOx)

Robertson described a risk score for the formation of calcium oxalate stones and observed that a PSF(CaOx) score >0.5 was predictive for lithiasis [20]. Therefore, we used this cutoff to assess patients at risk of urolithiasis. The ROC curve for UOx/Creat had an AUC of 0.955 (95% CI 0.930–0.980) for PSF(CaOx) >0.5. Table 3 presents the clinical PSF(CaOx) implications for different UOx/Creat thresholds, including the percentage of patients requiring confirmatory testing and the rates of cases with PSF(CaOx) >0.5 that would be missed (false negatives) and detected (true positives). In comparison to the data for hyperoxaluria diagnosis, a higher UOx/Creat threshold was associated with optimal screening performance for PSF(CaOx) >0.5: a threshold of 47.5 μmol/mmol would result in a false-negative rate of <2%, with 10% of patients requiring confirmatory testing.

**Table 3:** Clinical implications of different UOx/Creat thresholds for the PSF(CaOx) score

UOx/Creat threshold	Confirmatory test needed (%)	PSF(CaOX) result (%)			
		FN	TP	TN	FP
30 μmol/mmol	29.5	0.6	6.7	69.8	22.8
32.5 μmol/mmol	24.4	0.8	6.6	74.8	17.8
35 μmol/mmol	20.0	0.9	6.5	79.0	13.5
37.5 μmol/mmol	16.8	1.1	6.2	81.9	10.6
40 μmol/mmol	14.4	1.4	6.0	84.2	8.4
42.5 μmol/mmol	12.0	1.7	5.7	86.4	6.2
45 μmol/mmol	10.8	1.7	5.7	87.5	5.1
47.5 μmol/mmol	9.9	1.9	5.5	88.2	4.5
50 μmol/mmol	9.0	1.9	5.5	89.1	3.6
60 μmol/mmol	6.5	3.3	4.1	90.2	2.4
70 μmol/mmol	4.7	3.8	3.6	91.5	1.1
80 μmol/mmol	3.4	4.7	2.7	91.9	0.8

FN = false negative; FP = false positive; PSF(CaOX) = probability of calcium oxalate stone formation; TN = true negative; TP = true positive; UOx/Creat = urinary oxalate/creatinine ratio.

### 3.4 Diagnostic performance of UOx/Creat for crystalluria

The AUC for UOx/Creat prediction of whewellite crystalluria was 0.74 (95% CI 0.61–0.88). Table 4 summarizes the clinical impact of various UOx/Creat thresholds for whewellite crystalluria, including the proportion of patients requiring confirmatory testing and the rates of false-negative and true-positive results. As for PSF(CaOx) >0.5, a higher UOx/Creat threshold improves the screening performance for whewellite crystalluria: a threshold of 47.5 μmol/mmol limits the false-negative rate to <2%, with approximately 10% of patients needing confirmatory testing.

**Table 4:** Clinical implications of different UOx/Creat thresholds for crystalluria

UOx/Creat threshold	Confirmatory test needed (%)	Crystalluria result (%)			
		FN	TP	TN	FP
30.0 μmol/mmol	28.9	1.2	2.9	69.9	26.0
32.5 μmol/mmol	24.1	1.2	2.9	74.7	21.2
35 μmol/mmol	19.9	1.3	2.8	78.8	17.2
37.5 μmol/mmol	16.9	1.5	2.6	81.5	14.2
40 μmol/mmol	14.7	1.5	2.6	83.9	12.1
42.5 μmol/mmol	12.4	1.7	2.3	85.9	10.0
45 μmol/mmol	11.3	1.7	2.3	86.9	9.0
47.5 μmol/mmol	10.3	1.9	2.2	87.8	8.1
50 μmol/mmol	9.3	1.9	2.2	88.8	7.1
60 μmol/mmol	6.7	2.2	1.9	91.1	4.8
70 μmol/mmol	4.9	2.6	1.5	92.4	3.5
80 μmol/mmol	3.5	2.9	1.2	93.6	2.3

FN = false negative; FP = false positive; TN = true negative; TP = true positive; UOx/Creat = urinary oxalate/creatinine ratio.

The influence of the cost associated with false-negative results on UOx/Creat threshold selection for diagnosis of hyperoxaluria, PSF(CaOx) >0.5, and whewellite crystalluria is detailed in Supplementary Table 1.

## 4 Discussion

The aim of our study was to evaluate the performance of UOx/Creat in identifying hyperoxaluria and surrogates for associated complications in a population evaluated for the risk of urolithiasis.

In 2021, a consensus conference underscored the importance of 24-h urine analysis in diagnosing and managing calcium-containing stones, prescribing medications, offering dietary advice, and ensuring treatment adherence, although 24-h urine collection is not frequently performed, as indicated by rates as low as 7.4–13% for patients at risk of urolithiasis [9,[23], [24], [25]]. Collection of 24-h urine samples presents practical challenges for patients, as it requires strict adherence over an entire day. This can be difficult to integrate into daily routines because of work obligations, busy schedules, and other constraints. Consequently, 24-h urine samples are frequently incomplete, improperly collected, or unrepresentative of typical daily activities, as patients often perform collection on atypical days with lower activity levels, such as weekends, holidays, and days off work. The failure rate for complete 24-h urine collection ranges from 6% to 47% in the literature [26], and was 38% in our study. A urinary spot test is significantly easier to perform and offers patients lower time and logistical constraints, and thus appears to be a more feasible and pragmatic approach for screening patients at risk. Despite these advantages, it is essential to recognize that a properly collected 24-h urine sample remains the gold standard for diagnosis and management. In cases with an abnormal screening result for a spot urine test, confirmatory analysis of 24-h urine should be performed to ensure accurate diagnosis and tailored management for the patient.

Various studies have explored different ratios for detection of hyperoxaluria. Itami et al [27] investigated the correlation between spot UOx/Creat values and 24-h urinary oxalate excretion in children with and without primary hyperoxaluria. The authors observed high correlation of these parameters in morning first-voided urine (*r* = 0.996) [27]. Conversely, Hashmi et al [11] found poor correlation between UOx/Creat and 24-h oxaluria (*r* = 0.289) in adult patients with kidney stones, possibly because of incomplete 24-h urine collection and a limited number of hyperoxaluria cases. The authors reported sensitivity of 83.3%, specificity of 17.8%, a positive predictive value of 9.8%, and a negative predictive value of 90.9% for UOx/Creat cutoffs of 33 μmol/mmol for men and 45 μmol/mmol for women. Hong et al [28] investigated correlations and agreement between solute/creatinine ratios for 24-h and early-morning spot urine samples in 30 urolithiasis patients undergoing metabolic evaluation. The authors found poor correlation (*r* = 0.352) and agreement levels, possibly because they did not take sex disparity for the UOx/Creat ratio into account [28].

The differences observed across studies may be attributable to several factors. First, the populations investigated in these studies differed from our cohort, which may have an influence on the findings. In addition, the sample sizes were smaller in these studies, in contrast to our larger cohort, which potentially enhances the robustness of our results. Finally, our decision to exclude patients with unreliable 24-h urine sample collection—defined on the basis of creatinine levels—probably minimized variability and enhanced the accuracy of our analysis, further explaining the discrepancies observed. Clifford-Mobley et al [12] examined UOx/Creat in primary hyperoxaluria and found moderate correlation (*r* = 0.63) with 24-h oxaluria, akin to our findings, possibly because they used the UOx/Creat ratio for 24-h urine but with less rigorous assessment of the completeness of sample collection. Notably, the authors identified the 95th UOx/Creat centile as 39 μmol/mmol for their overall cohort, 33 μmol/mmol for men, and 45 μmol/mmol for women. These values correspond to the upper bounds for hyperoxaluria according to ERKNet recommendations [16] and are close to our optimal cutoff point for hyperoxaluria diagnosis identified via ROC analysis when the cohort was split into male and female groups (data not shown). In addition, day-to-day variation in oxalate excretion within an individual is higher for UOx/Creat (79%) than for 24-h urine oxalate (43%) [29]. Therefore, we observed that the UOx/Creat ratio is not sufficient to establish or refute a diagnosis of hyperoxaluria.

In the present study, we evaluated the UOx/Creat ratio in a large cohort encompassing diverse types of urolithiasis. A novel aspect of our study is the comparison of UOx/Creat to surrogate markers of lithiasis risk such as the PSF(CaOx) score [20] and the presence of whewellite crystalluria. Our UOx/Creat screening threshold (35–50 μmol/mmol) was associated with a high negative predictive value for these surrogate markers (92–99%), indicating satisfactory screening performance. Despite variations in the overall diagnostic performance for the PSF(CaOx) score and whewellite crystalluria because of differences in the nature of their measurement and the origin of the urine sample, the overall consistency of the UOx/Creat performance suggests robustness and reliability in identifying individuals at higher risk of hyperoxaluria-related complications.

Our study has significant clinical implications in terms of the potential to identify patients at elevated risk of hyperoxaluria-related complications who should undergo full 24-h urine evaluation for hyperoxaluria diagnosis and assessment of lithiasis risk. For example, the UOx/Creat ratio could be used as a screening tool to identify bariatric surgery patients who require a proper evaluation of lithiasis evaluation to mitigate risk factors for oxalate nephropathy and kidney stone formation in this vulnerable population.

Despite its contributions, our study has limitations. First, we used a common enzyme method to measure oxaluria. However, several analytical methods are available to quantify urinary oxalate (enzyme kits, chromatography methods coupled to mass spectrometry) [6,13,30] and the variability among the methods means that there are difference in values interpreted as being normal. Therefore, the UOx/Creat values from our study are valid for methods that have the same normal range as our method. If the normal range differs, the ratio must be recalculated. Moreover, we could have enhanced the performance and robustness by considering sex to select the screening ratio, but we opted for simplicity to facilitate easier implementation in clinical practice. However, this approach could lead to higher false-positive rates in the female population. Another notable limitation is the extrapolation of prognostic value from just one crystalluria sample; multiple assessments with a crystalluria index >0.50 (number of positive urine samples/number of samples examined) provide more accurate prognostic value, as described by Daudon et al [21]. In addition, measurement of the UOx/Creat ratio in the urine sample collected for 24-h analysis may introduce reproducibility issues. Use of an overnight urine sample, as suggested by Rodriguez et al [31] may be useful, as the sample from this time period contributes most to the variability observed in the supersaturation of 24-h urine. The retrospective, single-center design of this study is a limitation that restricts the generalizability of the findings, as they may not fully reflect broader multicenter populations or different health care settings. Given that the prevalence of hyperoxaluria in our population was 19%, the threshold must be adapted for other contexts, as lower prevalence could significantly increase the number of false-positive results. These limits highlight the need for further validation studies; our aim is to validate this ratio for urine samples from a bariatric surgery population.

## 5 Conclusions

In conclusion, our study underscores the practicality and efficiency of the UOx/Creat ratio as a screening tool for hyperoxaluria-related complications. We identified UOx/Creat ratio thresholds to help biologists and clinicians in interpreting oxaluria results. Nevertheless, further research is needed to overcome the limitations identified and validate the performance marker in an external cohort to confirm its clinical applicability and efficacy.

  ***Author contributions:*** Pierre Letourneau had full access to all the data in the study and takes responsibility for the integrity of the data and the accuracy of the data analysis.

  *Study concept and design*: Letourneau, Lemoine.

*Acquisition of data*: Machon, Poussineau.

*Analysis and interpretation of data*: Letourneau, Lemoine, Cabezas, De Mul.

*Drafting of the manuscript*: Letourneau, Lemoine.

*Critical revision of the manuscript for important intellectual content*: Machon, Cabezas, De Mul, Derain Dubourg, Bacchetta, Abid, Acquaviva.

*Statistical analysis*: Letourneau.

*Obtaining funding*: None.

*Administrative, technical, or material support*: Lemoine.

*Supervision*: Lemoine.

*Other*: None.

  ***Financial disclosures:*** Pierre Letourneau certifies that all conflicts of interest, including specific financial interests and relationships and affiliations relevant to the subject matter or materials discussed in the manuscript (eg, employment/affiliation, grants or funding, consultancies, honoraria, stock ownership or options, expert testimony, royalties, or patents filed, received, or pending), are the following: None.

  ***Funding/Support and role of the sponsor:*** This work was supported by Hospices Civiles de Lyon.

  ***Data sharing statement:*** The raw data and code are available on request.
