# Supplementary material for: Optimizing Screening Performance for the Risk of Hyperoxaluria and Urolithiasis Using the Urinary Oxalate/Creatinine Ratio: A Retrospective Analysis
Source: Eur Urol Open Sci. 2025 Mar 28;75:20–8. doi: 10.1016/j.euros.2025.03.003 (PMC11992522; doi:10.1016/j.euros.2025.03.003)
Supplement: Supplementary Data 1 [file mmc1.docx]

Supplementary material


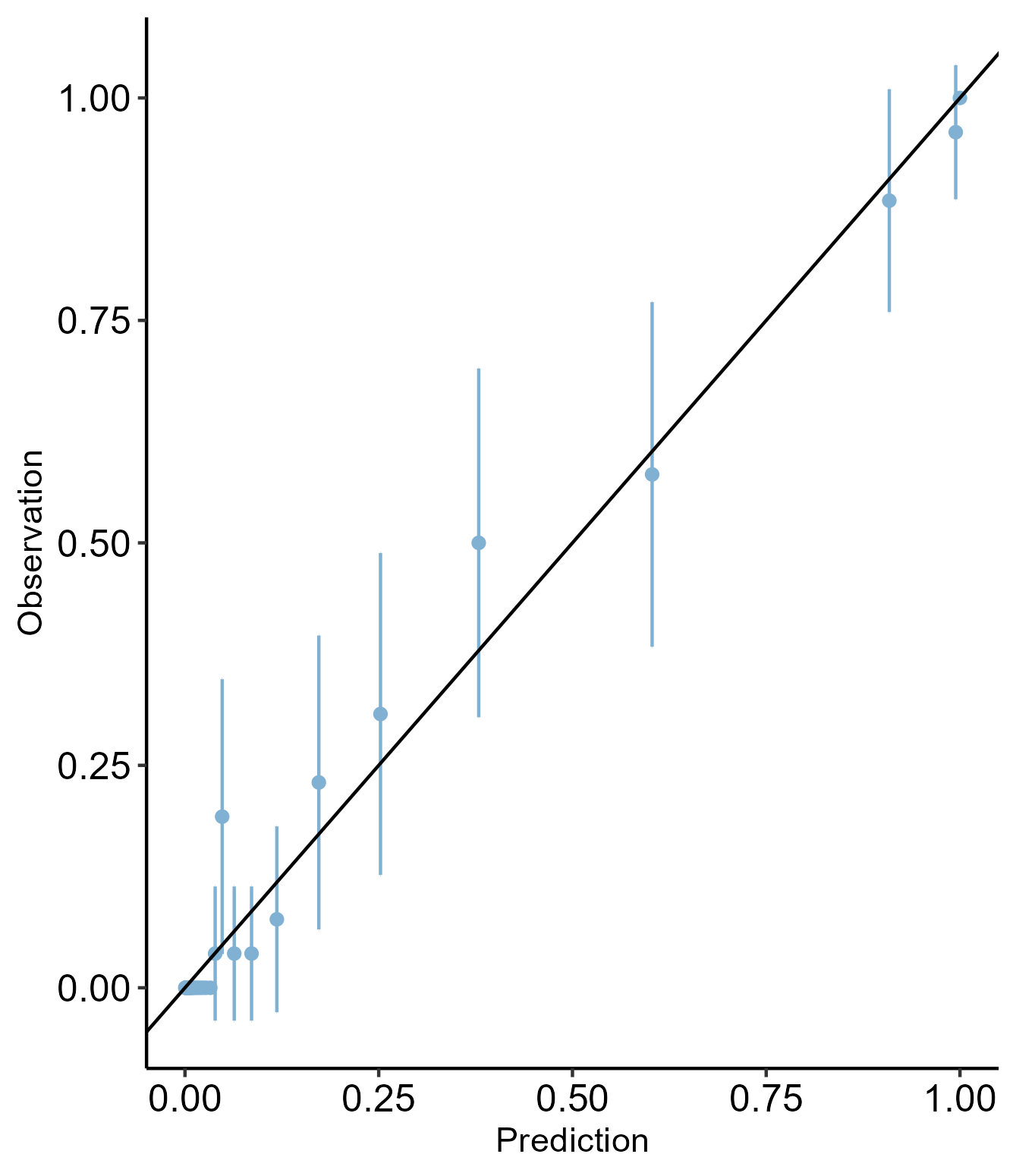


**Supplementary Figure 1. Calibration plot of the model predicting hyperoxaluria probability from the UOx/Creat ratio**

**Supplementary Table 1. UOx/Creat Ratio Thresholds Adjusted for Varying Costs of False Positives**

|  | **Cost of False positive** | **UOx/Creat Ratio Threshold (µmol/mmol)** |
| --- | --- | --- |
| **Hyperoxaluria** |  |  |
|  | 1 | 41.57 |
|  | 2 - 3 | 35.91 |
|  | 4 - 10 | **33.92** |
|  | 11 - 15 | 27.05 |
| **Crystalluria** |  |  |
|  | 1 | 128.68 |
|  | 2 - 3 | 106.95 |
|  | 4 - 15 | **57.92** |
| **PSF(CaOx) > 0,5** |  |  |
|  | 1 | 54.36 |
|  | 2 - 6 | **52.21** |
|  | 7 | 45.82 |
|  | 8 - 15 | 37.38 |
